# Rupture of the Uterus after Cæsarean Section

**Published:** 1909-08-07

**Authors:** 


### OBSTETRICS.

### RUPTURE OF THE UTERUS AFTER CÆSAREAN SECTION.

Any medical man may be confronted by the question: Should a patient, in the presence of absolute indications for Cæsarean section, be sterilised at the time of the first section? It is clear that many different factors are bound to be duly weighed and considered before so important a question can be answered justly. Should the patient strongly desire to be freed from the dangers she would otherwise be obliged to run, it is scarcely justifiable to refuse to take the steps necessary for doing so. On the other hand, the patient may be most desirous of having other children, and it is possible for her to have them if she runs the risks attendant on repeated Cæsarean sections. Is it justifiable to allow her to run these risks? It is clear that she runs not only the ordinary risks of anæsthetic and laparotomy, but also the danger that her uterus, already scarred, may rupture along the line of the previous incision. The question is: What is the risk that such rupture through a Cæsarean cicatrix will occur during a subsequent pregnancy?

Professor Brodhead, of New York, is amongst the recent writers on the subject, and he notes the wonderful difference in the frequency of rupture after the older forms of Cæsarean section as compared with the results following the more modern operations. In 1886 Krukenberg stated that after the old operation 50 per cent. of all cases resulted in rupture of the uterus during subsequent pregnancies. He reported 20 cases of rupture through the scar with a mortality of 50 per cent. On the other hand, Olshausen states that a given instance he speaks of was the only one of scar rupture in at least 120 Cæsarean sections.

Much has been written in regard to the cause of rupture, but briefly put there seem to be but two main factors in its ætiology. One is the natural weakness of a cicatrix, whether in the uterus, abdominal wall, perineum, or elsewhere, and the other is the invasion of the musculature of the uterus by decidual cells. If good approximation of the edges of the wound is secured, and interrupted sutures of chromic catgut are used, the result will be perfect as far as complete union of the parts is concerned; and in Professor Brodhead's opinion rupture of the Cæsarean scar is then very unlikely indeed to occur. He has analysed the available figures carefully, and he draws the following conclusions:—

1. That rupture of the uterus through the Cæsarean cicatrix is of rare occurrence.
2. That with prompt operative methods the mortality of such rupture is comparatively low.
3. That when pregnancy follows Cæsarean section the patient may be safely delivered again by section in a large percentage of cases.
4. That in repeating a Cæsarean section labour should be anticipated by a week or ten days.
5. That if Cæsarean section is to be repeated and labour sets in prior to the time fixed upon for the operation, the section should be performed as soon as possible after the onset of the labour pains.
6. That sterilisation may be done at the time of Cæsarean section if the patient so desires.
7. That in the case of rupture through the scar suture of the laceration has proved successful on occasion, but in some instances hysterectomy will be the method of choice.

In answer to our original question, it would seem that in the absence of any condition, such as carcinoma or fibroids, which in itself would indicate the removal of the uterus, the question of future pregnancy should be answered before the Cæsarean section is performed. The danger of repeated section and the possibility of rupture of the uterus during pregnancy and labour should be put before the patient plainly, and if she then elects to undergo repeated Cæsarean sections, she should be allowed to do so; indeed, she may do so without great risk.

